# Degree of Standardisation in Ceramic Gingival Systems

**DOI:** 10.3390/ma16206710

**Published:** 2023-10-16

**Authors:** Alejandra Díaz Hernández, Ana María Martín Casado, Miguel Gómez-Polo, Alicia Celemín Viñuela, Cristina Gómez-Polo

**Affiliations:** 1 Department of Prosthodontics, Faculty of Dentistry, University Complutense of Madrid, 28040 Madrid, Spain; alejandradiazhe@gmail.com (A.D.H.); miguelodont@hotmail.com (M.G.-P.); acelemin@odon.ucm.es (A.C.V.); 2 Department of Statistics, Faculty of Medicine, University of Salamanca, 37007 Salamanca, Spain; ammc@usal.es; 3 Department of Surgery, Faculty of Medicine, University of Salamanca, 37007 Salamanca, Spain

**Keywords:** gingival colour, spectrophotometry, pink ceramic samples, CIELAB colour space, gingival shade guide, gingival acceptability threshold

## Abstract

No gingival shade guide exists that can be used as a ‘gold standard’ in gingival shade selection. This research, therefore, aimed to determine whether comparable results in subjective gingival shade selection can be achieved using basic gingival colours produced by distinct manufacturers. It also aimed to explore how coverage of the colour space is affected by mixing these basic colours to create additional shades. To achieve these objectives, the basic gingival colours of three ceramic systems (Heraceram, Kulzer, Madrid, Spain; Vita VM9, Vita Zahnfabrik, Bad Säckingen, Germany; IPS Style, Ivoclar, Schaan, Liechtenstein) were analysed. The colour systems were expanded by creating porcelain gingival samples, whose colours were obtained by mixing the basic colours, altering each mixture by increments of 10%, and respecting the numerical order used by manufacturers to identify the colours. The colour coordinates of the basic and additional colours were recorded using spectrophotometry, and the intra- and inter-system colour differences were calculated using the Euclidean (ΔE_ab_) and CIEDE2000 (ΔE_00_) formulae. None of the basic colours in the three systems, despite their similar nomenclature, were found to be interchangeable (the colour differences exceeded the gingival acceptability threshold: ΔE_00_ 2.9 units). The expanded gingival colour systems, with mixtures altered by 10% increments, notably increased the gingival colour space covered by the original systems. The authors concluded that there are clear differences between the basic gingival colours produced by distinct manufacturers using the same nomenclature. Ceramic samples produced by mixing basic gingival colours are a resource with the potential to improve subjective gingival shade matching.

## 1. Introduction

Attractive smiles provide increased aesthetic satisfaction, together with more positive assessments of social interactions, employment, intellect, and success [1,2]. A positive perception of dental colour is the most important factor in smiles [3,4], even more so than correct alignment and symmetry [5]. For an appealing smile, achieving harmony between ‘white dental aesthetics’ and ‘pink gingival aesthetics’ is necessary [6,7,8,9], since a large part of the population show gingival tissue when they smile [10]. Gingival aesthetics depend on the state of gingival health: the presence of gingival or periodontal pathologies can cause infectious problems or systemic health problems [11,12]. Subjective comparison of teeth with the physical shade tabs in dental shade guides remains the most common colour-selection method [13,14,15]. Spectrophotometers and colourimeters were developed to provide objective colour measurement with high levels of precision and accuracy, the aim being to eliminate the subjectivity inherent to shade selection. These electronic devices are primarily used to measure dental colour in chromatic research, rather than everyday clinical practice, due to the elevated cost and a lack of training in their use. This is despite the fact that dental shade guides only represent the middle third of the tooth [16]; physical shade tabs are not systematically placed according to their spatial location [17,18,19]; these tabs have no established colour coordinates in the CIELAB system [14,20,21]; no dental shade guide is identical to any other [22,23,24,25]; coverage of the dental colour space is incomplete [26]; and there is significant intra- and inter-observer variability [27,28,29]. Moreover, factors such as the observer’s age, experience, vision fatigue, and visual deficiencies [22,30] mean that shade matches are not reproducible and do not facilitate consistent results [31,32]. Despite such limitations, shade guides provide a quick, cheap method for assessing dental colour that has been used successfully in numerous studies [33,34,35,36,37,38,39].

Dental shade guides are currently indispensable for shade selection in direct clinical restorations, and communicating shades to laboratories for indirect prosthetic restorations. The ‘gold standard’ is the Vita Classical guide, which serves as an international reference for dental colour, increasing the efficiency of communication between dentistry professionals. The validity and accuracy of shade guides are vital [40,41,42,43,44], both for dental restorations, restoring gingival defects, and deficient edentulous sites [45,46,47,48,49]; however, gingival shade guides and their physical shade tabs are few in number and specific to each manufacturer [50,51]. No ‘gold standard’ exists [52,53], which prevents the development of a shared language on gingival colour. This makes gingival shade selection more difficult, prevents electronic devices from providing chromatic readings that align with physical shade tabs, and hampers communication between clinics and prosthetic laboratories [54].

Most manufacturers present the basic gingival colours with the letter G followed by a number (normally between one and eight) but there is no evidence of standardisation, whereby the colours produced by different manufacturers could be used interchangeably. This would ensure that a G2 tab, for example, would represent the same shade in any setting, as occurs in dental colour selection, where A2 colours are homogenous across manufacturers. To confirm whether this is the case, the present study’s analysis of the colour coordinates of distinct manufacturers’ gingival shades is vital. Producing mixtures of basic ceramic colours as a means to increase the range of gingival shades has been described in previous research [55]. This potentially improves the chances of achieving similitude between restorations and adjacent gingival colour [55]. The present study’s chromatic analysis of the expanded colour systems is necessary to confirm whether this method increases coverage of the gingival colour space, thereby offering improved shade-matching results.

To standardise colour measurement, the CIE (Commission Internationale de l’Eclairage) [56] developed the CIELAB colour system in 1976, which has become the universally accepted colour specification system [57]. The chromatic model has three colour coordinates: L* denotes the amount of white and black (0 indicating black and 100 white); a* describes the transition from green (negative values) to red (positive values); and b* shows the position between blue (negative) and yellow (positive). There are two formulae that are widely used in dentistry to quantify the difference between two colours [57]—the classical Euclidean formula:

$$∆E_{ab}={\left[{\left(∆L^{*}\right)}^{2}+{\left(∆a^{*}\right)}^{2}+{\left(∆b^{*}\right)}^{2}\right]}^{1/2}$$

and the CIEDE2000 formula:

(1) $$∆E_{00}={\left[{\left(\frac{∆L^{\prime }}{K_{L}S_{L}}\right)}^{2}+{\left(\frac{∆C^{\prime }}{K_{C}S_{C}}\right)}^{2}+{\left(\frac{∆H^{\prime }}{K_{H}S_{H}}\right)}^{2}+R_{T}\left(\frac{∆C^{\prime }}{K_{C}S_{C}}\right)\left(\frac{∆H^{\prime }}{K_{H}S_{H}}\right)\right]}^{1/2}$$

Of the two, the CIEDE2000 formula correlates more closely with visual perception [58,59,60,61].

This study’s primary objective is to chromatically describe and compare the basic colours of three ceramic gingival colour systems (Heraceram, Kulzer; Vita VM9; Vita-Zahnfabrik; IPS Style, Ivoclar-AG). Its secondary objective is to chromatically describe and compare the three aforementioned ceramic gingival colour systems when expanded with mixtures of the consecutively ordered basic colours, the mixtures differing by 10% increments.

This study’s first null hypothesis is that standardisation exists between the basic colours of the three commercial brands, meaning that they offer interchangeable gingival colours. Its second null hypothesis is that the ceramic gingival systems that have been enlarged with mixtures of consecutively ordered basic colours (differing by 10% increments) do not expand the prosthetic gingival colour space.

## 2. Materials and Methods

### 2.1. Sample Preparation and Colour Coordinate Recording

**Figure 1:**
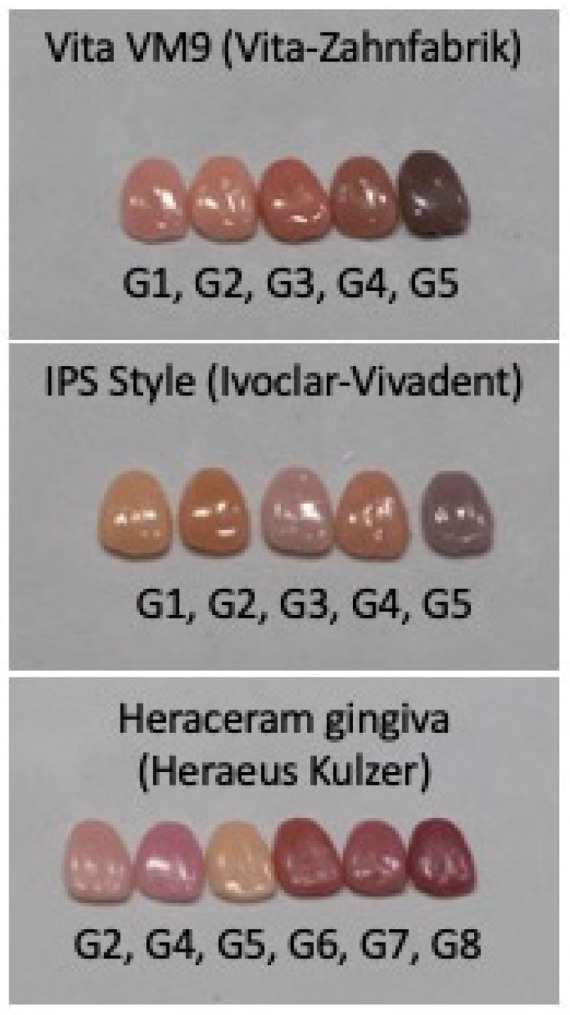
Original gingival colours used in this study, showing the manufacturers’ nomenclature of G (Gingival) + a distinct number for each colour.

Three original gingival colour systems were used as the basis for this study (Figure 1): (1) the Heraceram system (HK)—Kulzer GmbH—with six basic colours (G2, G4, G5, G6, G7, and G8); (2) the Vita VM9 system (VZ)—Vita-Zahnfabrik—with five basic colours (G1, G2, G3, G4, and G5); and (3) the IPS Style system (IV)—Ivoclar-AG—with five basic colours (G1, G2, G3, G4, and G5).

These original gingival colour systems were expanded by creating porcelain gingival samples, whose colours were obtained by mixing percentages of the basic colours in consecutive order, altering each mixture by increments of 10%, and respecting the numerical order used by the manufacturers to identify their basic colours. The basic colour mixtures were produced in the following way: G1 (90%) was mixed with G2 (10%), G1 (80%) with G2 (20%), G1 (70%) with G2 (30%), G1 (60%) with G2 (40%), G1 (50%) with G2 (50%), G1 (40%) with G2 (60%), G1 (30%) with G2 (70%), G1 (20%) with G2 (80%), G1 (10%) with G2 (90%), G2 (90%) with G3 (10%), and so on until G7 (20%) was mixed with G8 (80%), and finally G7 (10%) was mixed with G8 (90%). The expanded systems obtained and their composition were as follows: (1) a total of 6 basic colours and 45 mixed-colour samples—51 samples—for the Heraceram system (Kulzer, GmbH, Madrid, Spain); (2) a total of 5 basic colours and 36 mixed-colour samples—41 samples—for the Vita VM9 system (Vita-Zahnfabrik, Bad Säckingen, Germany); and (3) a total of 5 basic colours and 36 mixed-colour samples—41 samples—for the IPS Style system (Ivoclar-AG Schaan, Schaan, Liechtenstein) (Table 1).

All the ceramic gingival samples studied (basic colours and mixtures altered by 10% increments) were produced using a silicone template (New Architect wax-up assistant anterior Form B Large, SmileLine Europe GmbH) with the approximate dimensions of 10.6 mm × 61.6 mm × 33.2 mm, in a similar way to previous studies [55,61,62]. Porcelain dosifiers (Renfert) were used to accurately quantify the 10% increment alterations in the mixtures of basic colours.

The colour coordinates of all the samples used were recorded three times using the Spectroshade Micro spectrophotometer (MHT Optic Research AG, Niederhasli, Switzerland), after calibration, with daylight illumination (TLD 95/65 Phillips). The arithmetic mean was used in the statistical calculations. The Spectroshade spectrophotometer passed reliability–precision testing and had a configuration of 45° illumination and 0° observation.

### 2.2. Statistical Analysis

All of the statistical analyses were conducted using IBM’s SPSS software, version 26.0, including production of the three-dimensional figures and bar chart. Two three-dimensional figures were produced showing the colour coordinates: (1) for the original samples (the basic colours) of the three ceramic systems; (2) for the ceramic samples of the expanded systems. Using the Euclidean and CIEDE2000 formulae, the colour differences were calculated between the original ceramic samples of the three systems, and also between the ceramic samples of the expanded systems. To summarise these colour differences, basic descriptive indicators (minimum, maximum, mean, and standard deviation) were calculated. The colour differences obtained were compared to the 50:50% perceptibility and acceptability thresholds used in this study.

## 3. Results

### 3.1. Description and Comparison of the Basic Colours in the Three Ceramic Gingival Colour Systems

Table 1 shows the mean L*, a*, and b* colour coordinates for the basic gingival colours and the consecutive colour samples, mixed with 10% increment alterations, for the three ceramic systems examined (n = 133).

**Figure 2:**
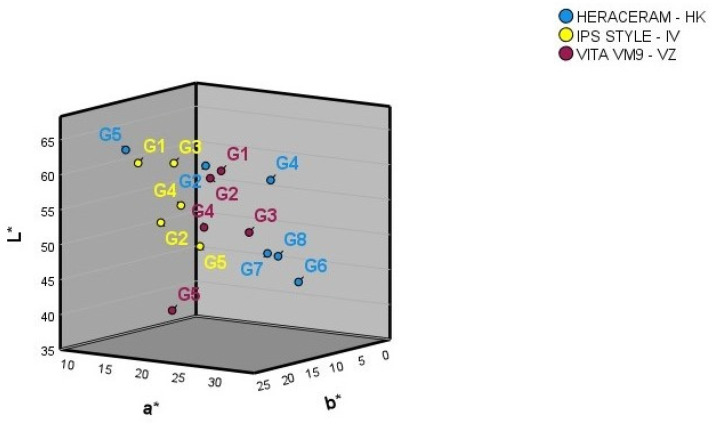
Three-dimensional representation of the basic gingival colours in the three ceramic systems examined.

Figure 2 shows the mean L*, a*, and b* colour coordinates for the basic gingival colours of the three ceramic systems. The numbers provided by the manufacturer for the basic gingival colours do not correspond with how any of the three colour coordinates are ordered spatially in any of the three ceramic systems examined. The spatial separation of the basic gingival colours is not equidistant in any of the three ceramic gingival colour systems.

Table 2 shows the colour differences between the basic colours of the three manufacturers. All the ΔEab colour differences between the basic colours of the HK and VZ shade guides are above the 50:50% acceptability threshold [55], meaning that none of the basic colours in these guides are interchangeable. The same is true for the basic colours of the HK and IV guides, except in the case of the HK G2 and IV G3 colours, whose colour difference is clinically acceptable (it is below the acceptability threshold of 4.1 but above the perceptibility threshold of 3.1) [55]. Finally, the ΔEab colour differences between the basic colours of the VZ and IV guides also fall above the acceptability threshold, meaning that the basic colours of these guides are not interchangeable either (Table 2). Further, there is no equivalence between the basic colours in the three ceramic systems (i.e., the basic gingival colours with a certain name in one of the ceramic systems are not interchangeable with those with the same name in the other two systems), given that the colour difference exceeds the acceptability threshold for gingival colour (Table 2) [55]. If the colour differences are measured using the CIEDE2000 formula, a comparison of the basic colours in the HK and VZ guides shows that only the HK G2 and VZ G1 colours have a colour difference below the acceptability threshold (2.9 [55]), although the difference is perceptible (above 2.1, the perceptibility threshold [55]). The same is true of colours HK G5 and IV G1.

Table 3 shows the colour differences (Euclidean formula—ΔEab; CIEDE2000 formula—ΔE00) for the basic-colour samples in each of the three gingival colour systems examined.

As can be seen in Table 3, all the colour differences exceed the 50:50% perceptibility and acceptability thresholds: colour differences that can be perceived/accepted by 50% of the observers (3.1 and 4.1, respectively, for ΔEab; 2.1 and 2.9, respectively, for ΔE00) [55]. The only exception is the colour difference between G7 and G8 in the Heraceram system (HK). Moreover, as the difference between the numbers identifying the colours increases, the colour differences do not increase proportionally in any of the three systems.

### 3.2. Description and Comparison of the Three Ceramic Gingival Colour Systems when Expanded with Basic-Colour Mixtures

**Figure 3:**
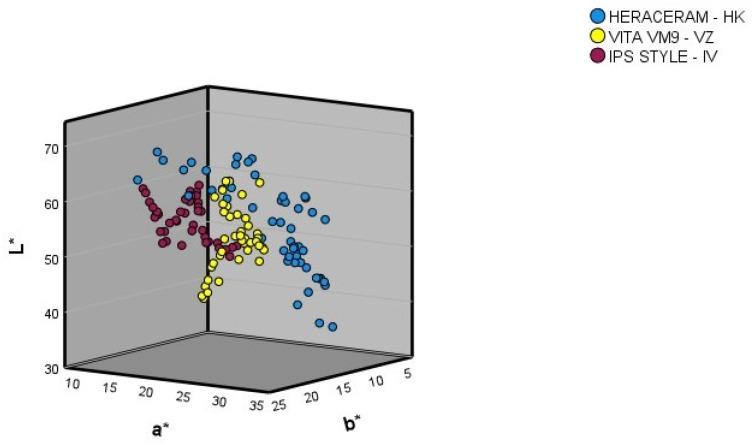
Three-dimensional representation of the gingival colours (basic colours and mixtures of the basic colours in 10% increments).

Figure 3 shows the L*, a*, and b* coordinates in the colour space for the gingival colours of the samples in the three expanded systems (basic colours and colour samples mixed in 10% increments).

Comparing the ranges of the L*, a*, and b* coordinates of the basic colours, and of the colour samples obtained through the mixtures produced in 10% increments (Table 1), the following observations can be made.

After expansion of the Heraceram system (Kulzer): (1) the range of the L* coordinate increased from 45.03–63.27 to 36.90–67.33, meaning that it contained both darker and lighter colours than those present in the original system; (2) the range of the a* coordinate increased from 13.87–31.30 to 13.63–32.90, meaning it contained redder colours than those present in the original system; (3) the range of the b* coordinate increased from 6.27–20.80 to 5.33–20.80, meaning it contained bluer colours than those present in the original system.

After expansion of the Vita VM9 system (Vita-Zahnfabrik): (1) the range of the L* coordinate increased from 37.83–60.50 to 37.83–62.83, meaning that it contained lighter colours than those present in the original system; (2) the range of the a* coordinate increased from 11.77–29.90 to 11.77–30.37, meaning it contained redder colours than those present in the original system; (3) the range of the b* coordinate increased from 8.13–20.10 to 8.13–20.90, meaning it included colours containing more yellow than those present in the original system.

After expansion of the IPS Style system (Ivoclar-AG): (1) the range of the L* coordinate increased from 46.57–62.30 to 46.15–63.00, meaning that it contained lighter colours than those present in the original system; (2) the range of the a* coordinate increased from 13.17–21.33 to 13.17–23.30, meaning it contained redder colours than those present in the original system; (3) the range of the b* coordinate increased from 4.57–24.87 to 4.57–25.67, meaning it included colours containing more yellow than those present in the original system.

Table 4 shows the descriptive indicators of all the colour differences, according to both formulae, for all the pairs of ceramic gingival samples, comprising basic colour mixtures altered by 10% increments.

**Figure 4:**
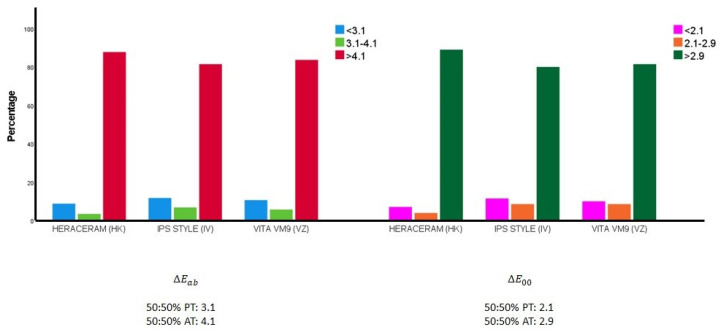
Distribution of the colour differences between all the pairs of samples in the expanded ceramic systems in relation to the 50:50% perceptibility (50:50% PT) and acceptability thresholds (50:50% AT) [55].

Figure 4 illustrates how the colour differences between the samples from the gingival system expanded with colour mixtures (in 10% increments) are distributed in relation to the 50:50% perceptibility and acceptability thresholds.

When the colour difference was calculated using the classic Euclidean formula: there were 112 pairs of samples (8.78% of the pairs) in the expanded HK system that were indistinguishable for 50% of observers, given that their colour differences fell below the 50:50 perceptibility threshold (3.1 units) [55]; there were 87 such pairs of samples (10.61%) in the expanded VZ system; while, in the expanded IV system, the number of sample pairs that were indistinguishable for 50% of observers was 96 (11.71%). When the colour difference was calculated using the CIEDE2000 formula: there were 90 pairs of samples (7.06% of the pairs) in the expanded HK system that were indistinguishable for 50% of observers, given that their colour differences fell below the 50:50 perceptibility threshold (2.1 units); there were 82 such pairs of samples (10.00%) in the expanded VZ system; while, in the expanded IV system, the number of sample pairs that were indistinguishable for 50% of observers was 94 (11.46%).

## 4. Discussion

### 4.1. Null Hypotheses

Based on these results, the first null hypothesis should be rejected, given that there is no chromatic standardisation of the basic gingival colours included in the pink ceramic systems examined: in the vast majority of cases, the colour differences exceed the clinical acceptability threshold (Table 2). Consequently, there are no gingival colours that may be used as a shared reference resource, despite the use of the same names to identify them. In other words, a G4 colour is distinct in each of the colour systems studied. It is not possible to select, communicate, or reproduce gingival colours precisely using a gingival shade guide, as each gingival colour has distinct colour coordinates. This is not the case for dental colour: there are no distinct dental shades with the same name, and a shared, universal language exists. A recent study on dental shade guides showed that the Vita Classical and Vita 3D Master guides [44] could be used interchangeably, with a high level of precision for different batches, and satisfactory scores for accuracy; however, the colour differences between distinct basic gingival colours produced by the same manufacturer do exceed the clinical acceptability threshold, which is a desirable characteristic for subjective gingival shade selection (Table 3). Ideally, these colour differences between each of the manufacturer’s consecutive basic colours should be homogeneous and equidistantly spaced in the three-dimensional colour space, which is not the case.

The second null hypothesis should also be rejected, since the creation of new ceramic samples in the form of basic-colour mixtures, altered by 10% increments, notably expands the colour space covered. This provides a wider range of options with which to achieve colour similitude when making subjective visual comparisons. It remains to be confirmed whether all the basic colours (n = 6 in HK; n = 5 in IV and HK) and all the 10% gingival colour mixtures (n = 45 in HK; n = 36 in IV and VZ) are representative of and useful for the colour space of gingiva, both in its natural condition and when signs of inflammation are present.

### 4.2. Strengths and Limitations of the Research

The main strengths of this study lie in its examination of the three most frequently used ceramic systems, as well as the percentage of the colour space covered by the mixtures produced in 10% increments, which gives an insight into the range of colour coordinates each colour system could offer, enabling future comparisons with gingival colour samples from various populations. One of the few spectrophotometers able to record gingival colour coordinates is the Spectroshade Micro (MHT Optic Research AG), which explains its extensive use [54,63,64,65,66,67,68]. The advantages of this spectrophotometer include its large aperture, enabling it to capture the entirety of the object being measured, as well as its near-perfect scores for repeatability and reproducibility [51], and the fact that it is unaffected by metamerism [69]. Despite these advantages, the ’edge loss phenomenon’ does occur, and this could have been minimized by using spectroradiometry [44]. Additional weaknesses include the fact that only one sample was produced for each gingival colour examined and that neither the thickness nor translucency parameters of the gingival shade tabs were taken into consideration.

### 4.3. Potential Clinical Applications

The results of this present research cannot be directly compared with similar studies, as it is the first to put forward the hypotheses detailed above. In light of the low percentage of sample pairs that are indistinguishable to the human eye, it may be concluded that expanding the number of ceramic gingival samples by producing colour mixtures is justified. This means that a high percentage of ceramic colour samples exceed the clinical acceptability threshold, making them potentially valid for use in subjective visual comparisons. The best expanded gingival colour system is that produced by Kulzer, which includes the lowest percentage of indistinguishable ceramic colour samples (Figure 4). It would be necessary to evaluate the chromatic fit of the colour samples produced (n = 133), along with the natural gingival colour of distinct populations (Caucasian, Asian, etc.), in order to confirm the validity of that expanded system.

In aesthetic dentistry, reproducing natural gingival colour through the various biomaterials in use has proved a considerable challenge in restorative and prosthetic procedures. This is particularly true in clinical situations in which there is extensive gingival exposure related to Miller class III and IV defects [46], bone defects or resorption in the anterior region, traumatic root extractions, or gingival recessions [70]. The epithelial characteristics of the gingival tissue, its anatomical scale, degree of vascularisation, and significant inter-subject and intra-subject variability [71,72,73,74] make emulating the adjacent gingival tissue through gingival restorations even more difficult.

The literature on gingival colour is not as developed as the research that has examined dental colour [52,75,76,77,78]. Most publications on gingival colour have focused on calculating the acceptability and perceptibility thresholds [55,79,80], designing optimal gingival guides using mathematical models of in vivo colour readings for healthy gingiva [50,53], studying the gingival colour coordinates of distinct populations [52,81,82], and the coverage errors of gingival shade guides [54,75,78,83,84,85,86]; however, there are no publications that tackle the fundamental issue of standardising gingival colours—even those with similar names—or that involve the production of new gingival colours using mixtures of basic gingival colours. This has led to significant limitations when it comes to communicating gingival colour, as there are no universally accepted reference resources for gingival colour, and the main manufacturers have developed colour systems without scientific publications explaining the rationale behind them or justifying the intra- and inter-system colour differences between the gingival colours. The letter G refers to ‘gingival’ and the number following it is not quantified in the manufacturers’ instructions, which simply state that a larger number alongside the letter G indicates a ‘more intense gingival colour’. This language appears similar to the nomenclature used by the Vita Classica dental shade guide (Vita-Zahnfabrik, Bad Sackinger, Germany). In 1998, Vita Zahnfabrik launched the Vita 3D Master guide, incorporating two improvements: it took into account all three dimensions of colour (lightness, chroma, and hue), as well as the colour space location [17,87,88,89,90,91,92]. Building on this experience in the dental colour field, it would be advantageous to conduct a rigorous, universally applicable homogenisation of gingival colour. A useful strategy for reproducing gingival appearance in detail is to provide intraoral photos of the adjacent gingiva alongside the physical gingival shade tabs.

Given that the type of shade guide can have a decisive influence on colour reproduction during the process of manufacturing a restoration, and that the chosen physical shade tab is considered the objective colour of the final restoration [44], establishing a universal gingival colour model that can be used for reference is necessary. It is of fundamental importance to have in place a standardised gingival shade guide that is well-designed in mathematical terms, takes into account the gingival acceptability/perceptibility thresholds, and is able to satisfactorily cover the natural gingival colour space. If no solid chromatic foundations are put in place for gingival colour, dentists will be limited to conducting shade selection that is contingent on the manufacturer of the chosen restoration material. It is in the dental sector’s interest to move forward in this area, so as to avoid future problems with communicating gingival colour. Measuring and reproducing gingival colour as objectively as possible is one of restorative dentistry’s greatest current challenges. It would be of great benefit for dentists and laboratory technicians to have standardised, valid reference resources for communicating and reproducing gingival colour, which are not dependent on the material and manufacturer used.

## 5. Conclusions

The present study shows that the basic colours of the ceramic systems examined are not standardised, despite their similar or identical nomenclature, since the colour differences exceed the gingival clinical acceptability thresholds. It also demonstrates that basic gingival colour mixtures, as produced in this study, expand the colour space and increase the likelihood of achieving colour similitude in subjective shade selection. This advance could contribute to developing gingival physical shade tabs that are of greater clinical use in the future. These results reveal the need to restructure the nomenclature and system employed using gingival shade guides, so as to ensure effective measurement, communication, and production at each stage of the productive process.

Future research should consider expanding the range of gingival materials, covering a larger number of manufacturers, and producing mixtures containing distinct colour percentages, in order to move towards securing a valid, homogenised set of gingival colours.

## Figures and Tables

**Table 1:** Colour coordinates for the ceramic samples in the gingival colour systems that were expanded with mixtures of basic colours, altered in 10% increments (HK—Heraeus Kulzer; VZ—Vita-Zahnfabrik; IV—Ivoclar-AG).

*HERACERAM (HK)*				*VITA VM9 (VZ)*				*IPS STYLE (IV)*			
	L*	a*	b*		L*	a*	b*		L*	a*	b*
**G2**	60.07	18.77	11.83	**G1**	60.40	22.93	14.93	**G1**	62.30	17.37	23.55
**90G2+10G4**	60.20	16.37	9.43	**90G1+10G2**	62.60	21.33	15.07	**90G1+10G2**	63.00	17.03	23.57
**80G2+20G4**	65.03	18.03	10.07	**80G1+20G2**	62.83	21.50	15.80	**80G1+20G2**	60.87	18.23	23.93
**70G2+30G4**	64.27	19.37	9.90	**70G1+30G2**	57.77	24.53	17.50	**70G1+30G2**	59.43	19.57	24.27
**60G2+40G4**	64.60	19.20	9.13	**60G1+40G2**	61.90	23.20	12.50	**60G1+40G2**	60.27	19.27	24.60
**50G2+50G4**	57.37	22.57	8.43	**50G1+50G2**	62.23	23.23	18.30	**50G1+50G2**	59.10	19.30	24.10
**40G2+60G4**	57.17	23.13	8.43	**40G1+60G2**	59.83	24.77	19.43	**40G1+60G2**	58.43	19.33	24.37
**30G2+70G4**	55.87	24.03	7.43	**30G1+70G2**	58.23	25.53	19.80	**30G1+70G2**	58.83	19.37	23.87
**20G2+80G4**	55.03	25.00	6.37	**20G1+80G2**	59.17	25.20	20.33	**20G1+80G2**	56.17	20.53	24.87
**10G2+90G4**	57.43	23.97	6.23	**10G1+90G2**	61.47	23.47	19.80	**10G1+90G2**	54.47	21.53	25.67
**G4**	57.70	23.90	6.27	**G2**	60.50	24.87	20.10	**G2**	54.53	21.33	24.87
**90G4+10G5**	53.57	25.83	5.33	**90G2+10G3**	60.03	25.13	19.77	**90G2+10G3**	56.00	20.53	24.23
**80G4+20G5**	58.20	22.93	8.57	**80G2+20G3**	55.50	27.93	20.90	**80G2+20G3**	56.27	23.30	23.13
**70G4+30G5**	61.80	19.73	9.27	**70G2+30G3**	56.17	27.87	20.73	**70G2+30G3**	56.70	19.27	22.00
**60G4+20G5**	63.90	18.40	10.73	**60G2+40G3**	58.20	27.53	19.63	**60G2+40G3**	56.73	18.87	20.57
**50G4+50G5**	58.40	19.00	12.77	**50G2+50G3**	57.00	28.07	19.83	**50G2+50G3**	57.57	17.93	18.77
**40G4+60G5**	63.50	17.13	13.87	**40G2+60G3**	54.53	29.57	20.30	**40G2+60G3**	56.97	17.57	17.67
**30G4+70G5**	65.17	16.17	15.07	**30G2+70G3**	54.00	30.27	20.67	**30G2+70G3**	58.67	16.00	15.80
**20G4+80G5**	59.73	17.13	16.63	**20G2+80G3**	55.57	28.93	19.40	**20G2+80G3**	58.20	16.23	15.40
**10G4+90G5**	67.33	13.63	17.47	**10G2+90G3**	53.07	30.37	20.03	**10G2+90G3**	59.53	15.23	14.20
**G5**	63.27	13.87	20.80	**G3**	53.33	29.90	19.73	**G3**	60.00	14.77	12.30
**90G5+10G6**	66.33	15.17	18.33	**90G3+10G4**	54.80	29.07	19.50	**90G3+10G4**	59.13	15.30	13.27
**80G5+20G6**	64.20	16.27	16.40	**80G3+20G4**	50.83	29.60	19.87	**80G3+20G4**	58.97	16.33	14.47
**70G5+30G6**	60.80	19.43	15.63	**70G3+30G4**	53.37	27.07	18.33	**70G3+30G4**	58.13	17.30	15.30
**60G5+40G6**	61.00	20.40	15.10	**60G3+40G4**	53.50	25.33	17.23	**60G3+40G4**	57.63	17.87	15.97
**50G5+50G6**	52.67	25.07	14.33	**50G3+50G4**	51.57	25.97	17.53	**50G3+50G4**	57.17	18.80	16.57
**40G5+60G6**	55.10	25.93	12.37	**40G3+60G4**	52.53	25.00	17.00	**40G3+60G4**	57.37	18.80	17.03
**30G5+70G6**	54.00	26.87	11.83	**30G3+70G4**	53.27	25.40	17.77	**30G3+70G4**	55.47	16.70	17.97
**20G5+80G6**	57.83	23.53	13.97	**20G3+80G4**	53.40	23.37	16.50	**20G3+80G4**	54.73	20.93	18.80
**10G5+90G6**	55.37	25.40	13.03	**10G3+90G4**	49.47	24.30	17.00	**10G3+90G4**	58.97	15.90	14.83
**G6**	45.03	31.30	11.73	**G4**	52.10	20.80	15.23	**G4**	56.20	21.17	20.50
**90G6+10G7**	50.50	29.00	12.37	**90G4+10G5**	49.17	19.25	13.87	**90G4+10G5**	52.97	21.03	22.13
**80G6+20G7**	51.13	28.40	12.40	**80G4+20G5**	48.20	18.50	13.27	**80G4+20G5**	52.13	20.53	17.77
**70G6+30G7**	52.00	27.60	12.70	**70G4+30G5**	42.87	17.17	11.97	**70G4+30G5**	51.83	17.67	14.67
**60G6+40G7**	43.67	30.87	13.70	**60G4+40G5**	45.70	15.70	11.13	**60G4+40G5**	50.77	17.67	14.13
**50G6+50G7**	51.33	28.93	13.80	**50G4+50G5**	44.67	14.83	10.43	**50G4+50G5**	49.03	17.43	12.23
**40G6+60G7**	51.35	28.40	13.10	**40G4+60G5**	41.80	13.47	9.40	**40G4+60G5**	49.57	16.50	11.40
**30G6+70G7**	50.83	27.90	14.13	**30G4+70G5**	39.70	13.77	9.80	**30G4+70G5**	47.63	15.70	9.23
**20G6+80G7**	50.33	29.60	15.23	**20G4+80G5**	40.47	12.83	9.10	**20G4+80G5**	46.15	15.57	8.43
**10G6+90G7**	42.87	32.90	17.70	**10G4+90G5**	38.50	12.03	8.70	**10G4+90G5**	46.93	14.13	6.37
**G7**	49.47	29.37	15.17	**G5**	37.83	11.77	8.13	**G5**	46.57	13.17	4.57
**90G7+10G8**	49.07	29.80	14.57								
**80G7+20G8**	49.77	28.97	13.37								
**70G7+30G8**	47.97	30.43	13.50								
**60G7+40G8**	38.37	32.47	13.80								
**50G7+50G8**	45.77	30.67	12.23								
**40G7+60G8**	45.57	30.60	11.80								
**30G7+70G8**	45.30	30.23	11.07								
**20G7+80G8**	44.07	30.67	10.87								
**10G7+90G8**	36.90	32.07	11.30								
**G8**	48.90	29.90	13.80								

**Table 2:** (**a**) Colour differences, calculated using the Euclidean formula (ΔE_ab_), between the basic colours of the three ceramic gingival systems: Heraceram (HK), Vita VM9 (VZ), and IPS Style (IV); (**b**) Colour differences between the basic colours of the three ceramic gingival systems, calculated using the CIEDE2000 formula (ΔE_00_).

**(a)**	**ΔE_ab_**	**VZ G1**	**VZ G2**	**VZ G3**	**VZ G4**	**VZ G5**	**(b)**	**ΔE_00_**	**VZ G1**	**VZ G2**	**VZ G3**	**VZG4**	**VZG5**
	**HK G2**	5.20	10.29	15.22	8.90	23.61		**HK G2**	2.58	5.05	8.67	7.73	22.72
	**HK G4**	9.12	14.14	15.37	11.01	23.35		**HK G4**	6.57	9.44	9.22	8.76	21.33
	**HK G5**	11.17	11.36	18.89	14.28	28.50		**HK G5**	9.10	7.99	13.74	12.58	26.70
	**HK G6**	17.79	18.73	11.61	13.13	21.12		**HK G6**	16.01	16.89	9.91	9.77	12.68
	**HK G7**	12.69	12.89	6.00	8.96	22.24		**HK G7**	10.89	11.50	4.75	5.39	14.76
	**HK G8**	13.49	14.13	7.40	9.75	21.99		**HK G8**	11.66	12.47	5.82	6.27	14.49
	**ΔE_ab_**	**IV G1**	**IV G2**	**IV G3**	**IV G4**	**IV G5**		**ΔE_00_**	**IV G1**	**IV G2**	**IV G3**	**IV G4**	**IV G5**
	**HK G2**	12.01	14.40	4.03	9.79	16.32		**HK G2**	8.43	9.30	3.01	6.33	14.28
	**HK G4**	19.04	19.04	11.18	14.57	15.55		**HK G4**	14.44	13.41	8.43	10.47	12.88
	**HK G5**	4.56	12.19	9.15	10.17	23.30		**HK G5**	2.42	9.01	6.67	8.17	19.47
	**HK G6**	25.14	19.03	22.31	17.44	19.55		**HK G6**	21.43	15.32	17.53	14.68	10.20
	**HK G7**	19.46	13.58	18.23	11.87	19.58		**HK G7**	16.19	10.58	12.84	9.58	10.44
	**HK G8**	20.78	15.09	18.82	13.21	19.25		**HK G8**	17.25	11.73	13.59	10.66	10.19
	**ΔE_ab_**	**IV G1**	**IV G2**	**IV G3**	**IV G4**	**IV G5**		**ΔE_00_**	**IV G1**	**IV G2**	**IV G3**	**IV G4**	**IV G5**
	**VZ G1**	10.43	11.65	8.58	7.19	19.85		**VZ G1**	8.15	8.71	4.94	5.72	15.66
	**VZ G2**	8.45	8.42	12.77	5.69	23.92		**VZ G2**	6.39	7.04	6.18	4.55	17.14
	**VZ G3**	15.88	10.06	18.13	9.22	23.57		**VZ G3**	12.16	7.24	10.13	5.89	13.23
	**VZ G4**	13.60	9.96	10.36	6.69	14.23		**VZ G4**	11.54	6.39	8.27	5.16	9.50
	**VZ G5**	29.46	25.51	22.76	24.06	9.54		**VZ G5**	26.19	18.99	22.31	19.75	8.55

**Table 3:** Colour differences between the basic gingival colours of the three ceramic systems examined.

*Heraceram-Kulzer (HK)*			*Vita VM9-Vita Zahnfabrik (VZ)*			*IPS Syle-Ivoclar AG (IV)*		
Pair	ΔE_ab_	ΔE_00_	Pair	ΔE_ab_	ΔE_00_	Pair	ΔE_ab_	ΔE_00_
**G2-G4**	7.93	5.93	**G1-G2**	5.52	3.00	**G1-G2**	8.82	7.37
**G2-G5**	10.71	8.77	**G1-G3**	11.03	7.41	**G1-G3**	11.77	6.93
**G2-G6**	19.57	16.20	**G1-G4**	8.57	7.84	**G1-G4**	7.81	6.67
**G2-G7**	15.36	11.41	**G1-G5**	26.08	23.70	**G1-G5**	25.01	19.36
**G2-G8**	15.89	12.08	**G2-G3**	8.77	7.18	**G2-G3**	15.20	8.67
**G4-G5**	18.52	14.85	**G2-G4**	10.53	8.32	**G2-G4**	4.68	3.03
**G4-G6**	15.66	13.32	**G2-G5**	28.79	24.39	**G2-G5**	23.28	14.88
**G4-G7**	13.30	9.78	**G3-G4**	10.23	4.59	**G3-G4**	11.07	6.22
**G4-G8**	13.05	9.90	**G3-G5**	26.52	18.22	**G3-G5**	15.58	14.15
**G5-G6**	26.81	22.56	**G4-G5**	18.32	15.02	**G4-G5**	20.26	14.11
**G5-G7**	21.50	17.47						
**G5-G8**	22.64	18.46						
**G6-G7**	5.93	5.12						
**G6-G8**	4.60	4.13						
**G7-G8**	1.57	1.17						

**Table 4:** Colour differences (maximum, minimum, mean, and standard deviation) for the entire set of sample pairs in the three expanded ceramic systems, calculated using the Euclidean and CIEDE2000 formulae.

	Heraceram *Kulzer (HK)*		Vita VM9 *Vita-Zahnfabrik (VZ)*		IPS Style *Ivoclar-AG (IV)*	
	ΔE_ab_	ΔE_00_	ΔE_ab_	ΔE_00_	ΔE_ab_	ΔE_00_
Min	0.28	0.25	0.53	0.27	0.36	0.29
Max	36.11	32.79	28.84	25.95	25.42	19.86
Mean	12.22	9.95	11.50	8.74	9.10	6.47
SD	7.14	6.19	7.62	6.28	5.31	4.12

## Data Availability

Not applicable.
